# IMAGE: A New Tool for the Prediction of Transcription Factor Binding Sites

**Published:** 2008-10-03

**Authors:** R. Casilli, A. Marongiu, S. Melchionna, P. Palazzari, R. Paparcone, V. Rosato

**Affiliations:** 1 Ylichron Srl, ENEA Casaccia Research Center, Via Anguillarese 301, 00123 S. Maria di Galeria (Italy); 2 ENEA, Portici Research Center, Computing and Networks Service, Via, Vecchio Macello, 80055 Portici (Italy); 3 INFM-SOFT, Department of Physics, University of Rome “La Sapienza”, P.le A. Moro 5, 00186 Roma (Italy); 4 ENEA, Casaccia Research Center, Computing and Modelling Unit, Via Anguillarese 301, 00123 S. Maria di Galeria (Italy)

## Abstract

IMAGE is an application tool, based on the vector quantization method, aiding the discovery of nucleotidic sequences corresponding to Transcription Factor binding sites. Starting from the knowledge of regulation regions of a number of co-expressed genes, the software is able to predict the occurrence of specific motifs of different lengths (starting from 6 base pairs) with a defined number of punctual mutations.

## 1. Introduction

The discovery of Transcription Factor binding sites is still an open problem, as most of the softwares available to date have low predictive character, particularly for complex DNA (such as the human DNA). A novel method is proposed which overcomes some of the limitations affecting the existing prediction tools. Decoding the regulatory regions in DNA via the discovery of recurrent patterns is a major challenge in bioinformatics. The expression of a gene takes place when a region of the DNA sequence is transcribed into a RNA sequence, subsequently translated into the protein encoded by the gene. Transcription is initiated by one or more proteins called transcription factors (TF) binding to DNA. A TF recognizes a set of short nucleotide fragments called binding sites (BS), located within a “regulation region” typically up-stream from (often quite close to) the transcription start site, which then act as regulatory signals. The discovery of TF and BS in the regulation regions is a central issue in the post-genomic research. Computational methods seem, in this respect, to provide a useful approach to make “predictions” on the position of these entities, offering valuable insights for subsequent experimental studies [Tompa et al. 2005].

The problem of identifying BS can be formulated in simple terms by considering a set of genes regulated by the same TF (co-regulated genes). Typically, one assumes that the regulation regions are comprised within a few thousand nucleotides, upstream from the transcription start site. In this set, one seeks for one or more similar motifs, i.e. nucleotide patterns which are significantly over-represented. Recent findings [Prakash and Tompa, 2005] point out the higher phylogenetic conservation of the regulatory elements with respect to the surrounding non-functional sequence. The search for regulatory elements in terms of “signal finding” stems from the fact that, however, the putative signals usually present few mutations, insertions and deletions with respect to a consensus motif, i.e. phylogenetic conservation should allow to cope with a different DNA folding and, thus, consider the functional role played by mutations, insertions and deletions to accomodate the *structure* of the regulatory element.

When tackling the discovery of patterns of length *n* presenting only mutations from the consensus, an exhaustive search for all possible 4*^n^* mutations of a candidate motif becomes rapidly prohibitive, even on modern computers. The goal of detecting all possible over-represented patterns can be formulated as a multiple alignment problem, whose solution is known to be NP-complete [Jones and Pevzner, 2004]. In the past, several methods have been proposed to solve this challenging computational task (for an extensive review, see [Baldi and Brunak, 1998]). Exhaustive motif search algorithms have been proposed which rely on proper heuristics and pruning of the search space, such as the approach pioneered by Marsan and Sagot [Marsan and Sagot, 2000]. Different strategies are based on a statistical sampling of search space by ad-hoc Monte Carlo methods [Liu, 2002], such as the so-called Gibbs sampling method [Lawrence et al. 1993], or by maximization of proper scoring or likelihood functions. Other methods rely on diverse statistical models, such as detecting hidden Markov chains [Thijs et al. 2001].

A recent paper has assessed the performances of several computer programs, each operating with different heuristics, and found that the program Weeder [Pavesi et al. 2004], based on a quasi-exhaustive enumerative procedure, outperformed other methods [Tompa et al. 2005]. Overall, no method was found to have a correctness superior to 30%, in particular when analysing data sets relative to eucaryotic organisms. Therefore, despite the numerous available approaches and the scientific effort in this field, the detection of binding sites is still a partially unsolved problem.

In the present paper, we describe a strategy to discover binding sites inspired by a technique used for lossy image compression, known as vector quantization [Nasrabadi and King, 1988], and by analogous methods to identify genes with similar functions and reconstruct phylogenetic trees by clustering algorithms [Jones and Pevzner, 2004]. The central idea of our approach is to map all possible *n*-length substrings of a given DNA sequence into a properly defined *n*-dimensional space equipped with a distance measure which projects similar substrings, representing the same motif, into nearby points. Consequently, the goal of finding recurrent similar strings is shifted into the determination of highly clustered data points in a search space of high dimensionality.

We developed a fast and adaptive algorithm to detect clusters and cluster representatives. The latter are strings having a close resemblance to consensus motifs. The approach enables us to make an extensive search of clusters by automatically excluding a very large amount of strings which fall into the low-density regions of the search space. Notwithstanding the lack of strategies to provide optimal clustering solutions and the lack of a universal notion of what is a good cluster, our approach offers a number of advantages which we briefly enumerate. At first, the search method is based on a number of controlled heuristics allowing us to scan a large number of recurrent patterns with high efficiency. Secondly, the algorithm is sensitive to the choice of the starting conditions but samples extensively the clusters by running over a small number of initial conditions, so that the method proves convenient to investigate large and noisy data sets. Thirdly, a crucial benefit of such approach relies on the flexibility in choosing a convenient metrics in search space. In particular, while the classical definition of the motif finding problem is based on the notion of similarity between two motifs in terms of the Hamming distance [Jones and Pevzner, 2004], we will employ a wider definition by including the edit distance in the metrics. On the other hand, we will make minimal assumptions on the structure of the consensus motif, e.g. on the position of mismatches along the set of over-represented patterns. Given the above, the method is viable for use in different contexts of computational biology together with providing useful insight into the specific problem of predicting TF binding sites.

The proposed search algorithm allows to find a large number of over-represented strings with an affordable computing time (order of minutes for typical cases). The candidates are subsequently analyzed with standard indicators in order to assess their statistical significance, in particular when compared to a background sequence. We anticipate that, for the specific problem at hand, IMAGE provides a wealth of information, specifically a large number of recurrent patterns, i.e. high sensitivity to true positives, but with a somehow reduced specificity, so that the tool can be used either as is, or as a filtration step towards more TF-oriented, but more CPU-intensive, softwares.

## 2. Methods

Let us start describing our method by defining a few basic quantities. Given a string composed by *n* nucleotides *x* = (*x*_1_, …, *x_n_*) this is mapped onto a set of *n* coordinates, each defined on the discrete set {*A*, *C*, *G*, T}. The string *x* represents a point of coordinates (*x*_1_, …, *x_n_*) in the *n*-dimensional string space *N* = {*A*, *C*, *G*, *T*}*^n^*. We adopt the following encoding *e*:

$$\begin{array}{l} e(A)=\;<1000>,e(C)=\;<0100>,\\ e(G)=\;<0010>,e(T)=\;<0001>. \end{array}$$

The pattern *x* is expressed as a 4 × *n* matrix

(1) $$\omega_{l,a}(x)=\delta_{x_{1},a}$$

where *δ* is the Kronecker function, *a* = *A*,*C*,*G*,*T* and *l* = 1, …, *n*. Therefore, each string is represented as a point in the 4*n*-dimensional discrete space *N^D^* = {0, 1}^4^*^n^* subject to the constraints $\sum_{a=A,C,G,T}\omega_{l,a}=1$ for *l* = 1,*n*.

We use the Hamming distance between two points *x* and *y* as a measure of similarity between strings. The Hamming distance *d_H_*(*x*, *y*) quantifies the number of mismatches between two strings of length *n* by comparing the patterns letter by letter. In terms of matrix representation,

(2) $$d_{H}(x,y)=\frac{1}{2}\sum_{l=1}^{n}\sum_{a=A,C,G,T}\left|\omega_{l,a}(x)-\omega_{l,a}(y)\right|$$

Therefore, two strings with *l* mismatching characters have Hamming distance equal to *l*.

Given a text composed by the DNA sequence of length *L* (>> *n*), we consider all possible *L* − *n* + 1 substrings of length *n* obtained by shifting a window of size *n* over the text by one offset position. Each *n*-mer defines a point in *N^D^*. Moreover, the biological problem at hand is restated as the search for over-represented patterns occurring one or multiple times, with not more than *m* defects, in any of *K* distinct input DNA sequences. Given a set of *K* input DNA sequences with *l_i_* bases (*i* = 1, …, *K*), we search for the motifs which are mostly over-represented with respect to a predefined background distribution. A given *n*-mer is counted as an occurrence of the motif when the Hamming distance between the *n*-mer and the motif is smaller than *m*.

In order to quantify the mutual similarity among a group of *n*-mers we make use of the concept of profile matrix [Stormo, 2000], i.e. a 4 × *n* matrix, whose (*i*, *j*) element counts the frequency of occurrence of nucleotide *i* in position *j* of all strings. The matrix is further normalized along the columns $\left(\sum_{a=1}^{4}\omega_{l,a}=1\right)$ and thus its elements have values in the range [0,1]. Thus, by relaxing the condition on the discrete nature of *N* *^D^*, we consider the continuous space *N^C^* = [0, 1]^4^*^n^* whose elements have coordinates spanning the interval [0,1], still retaining the constraint $\sum_{a=A,C,G,T}\omega_{l,a}=1$ for *l* = 1,*n*.

We extend the definition for the metrics (2) to the continuous space. In particular, if one of the two matrices *ω* (*x*) is a discretized matrix in *N^D^* the metrics further simplifies to

(3) $$d_{H}(x,y)=n-\sum_{l=1}^{n}\omega_{l,x_{l}}(y)$$

being *x*_l_ the character in the *l*-th position of the *x* sequence.

Following [Stormo, 2000], the consensus pattern is defined as the string built from the profile matrix having in each position the nucleotide *a* corresponding to the largest value in the column. In other words, the consensus patterns is encoded by the discretized version of the profile matrix *Q: N^C^* → *N^D^* according to the expression

(4) $$\omega_{l,a}(Q(\hat{x})\equiv {\tilde{\omega }}_{l,a}=\{\begin{array}{cc} 1 & \text{if }\omega_{l,a}↔{\text{max}}_{k}\{\omega_{k,a}\}\\ 0 & \text{elsewhere} \end{array}$$

### 2.1. Definition of class representatives

Armed with a metrics in string space, we now face the problem of extensively searching clusters. Let us consider a sequence *s_x_* identifying *N = L* − *n* + 1 points *s_x_* *=* {*x*_1_, *x*_2_, …, *x_N_*} (each point represents a discretized profile matrix in *N^D^* ). Let us partition the points in *C* classes, each having a class representative (CR) *f̂* ∈ *N^C^* which operationally identifies the partitioning. The class *c_α_* is defined as the set of points having minimal Hamming distance from the *α*-th CR. Formally, the *C* class representatives {***f̂***_1_, …, ***f̂C***} induce a partitioning on *s_x_* given by

(5) $$s_{x}=\overset{C}{\underset{\alpha =1}{\cup }}c_{\alpha }\;\text{where }\left\{x_{i}\in c_{\alpha }↔\underset{\alpha }{\text{min}}d_{H}(x_{i},x_{\alpha })\right\}$$

The *C* class representatives {***f̂***_1_, …, ***f̂C***} are determined by solving the minimum problem

(6) $$\underset{\left\{\hat{x}\right\}}{\text{min}}\sum_{\alpha =1}^{C}\sum_{x_{i}\in {\hat{x}}_{\alpha }}d_{H}(x_{i},{\hat{x}}_{\alpha })$$

which can be formulated as a global minimum problem of a cost function. In geometric terms, a CR constitutes the centroid of points belonging to the class. The optimal partitioning is obtained when all centroids “fit” at best the classes to which they separately belong.

It should be noticed that the standard definition of the motif finding problem can be reframed as a median string problem [Jones and Pevzner, 2004], i.e. as the motif *x̃* solution of the following double minimization procedure

(7) $$\underset{\hat{x}}{\text{min}}\underset{\left\{x\right\}}{\text{min}}\sum_{t=1}^{K}d_{H}(x_{t},\tilde{x})$$

where {*x*} is any array of n-mers each placed on the *K* DNA sequences and, strickly speaking, *x̃* belongs to *N^D^*. Therefore, we reformulated the problem as a constrained search of an ensemble of, mutually exclusive, class representatives and, in case of a succesfull search, we should be able to find at least one class representative such that **f̂** = **x̃**. A further remark concerns if, in its original definition, the Hamming distance is a good definition. In particular, the idea of minimizing the sum of distances between instances and the motif may not work well if most of the positions not containing the motif are placed at random. In the present work, we decided to avoid the use of additional informations on the structure of the profile matrix in order to keep the method general.

To date, there is no known efficient (polynomial) method to solve the partitioning problem exactly (i.e. to locate the global minimum of the cost function), but some good clustering algorithms have been known for some time, such as the LBG algorithm in the image compression community [Linde et al. 1980], also known as the Lloyd k-Means algorithm [Jones and Pevzner, 2004] in computational biology. In this approach, an arbitrary partition of points is initially assigned by placing CRs at random and associating data points to each class. Next, the partition is improved by recomputing the new CRs corresponding to the current set of classes and the points are further redistributed among the classes, according to the just computed CRs. Therefore, at each step a new partitioning is reconstructed corresponding to the optimized position of the CRs. For the sake of clarity, we report the structure of the clustering LBG algorithm.

#### Input

1. training sequence *s_x_* = {*x*_1_, …, *x_N_*};
2. number of class representatives *C*;
3. tolerance threshold *ε*;

#### Output

1. set of *C* class representatives f̂^*^ = {*f̂*_1_^*^, …, f̂_C_^*^} which (nearly) solve the minimization problem
  $${\hat{x}}^{*}={\text{min}}_{\hat{x}}\left(\sum_{c_{r}}\sum_{x_{j}\in c_{r}}\left\|x_{j}-{\hat{x}}_{j}\right\|\right);$$

**Table:** 

**Begin algorithm**
**randomly select** an initial set of CR
*f̂*_1_^(0)^ = {*f̂*_1_^(0)^, …, *f̂_C_*^(0)^}; *k =* 0; *end = false*;
**while** not *end*
{
**Partition** the training sequence *s_x_* into *C* classes *c_r_*^(^*^k^*^)^ so that
***xj*** ∈ ***cr***^(^***k***^)^ ⇔ ***r*** = min***i*** ||***xj*** − ***f̂i***^(^***k***^)^||;
**Compute** the average distorsion ***D***(***f̂***^(^***k***^)^) associated to the array of CRs
${\hat{x}}^{\left(k\right)}:D({\hat{x}}^{\left(k\right)})=\frac{1}{N}\sum_{c_{r}}\sum_{x_{j}\in c_{r}}∥x_{j}-{\hat{x}}_{i}^{k}∥$
**If** (*k* > 0) Then
{
$\mathrm{If}\;\left(\frac{D({\hat{x}}^{\left(k\right)})-D({\hat{x}}^{\left(k-1\right)})}{D({\hat{x}}^{\left(k-1\right)})}\right)<ɛ$
Then *end* = *true*
}
**Compute** for each class *c_r_* the new CR as mean value of points belonging to *c_r_*, i.e.
${\hat{x}}_{r}^{\left(k+1\right)}=\frac{1}{\left\|c_{r}\right\|}\sum_{x_{j}\in c_{r}}x_{j}\;\;\;(r=1,\ldots ,C)$
*k* = *k* + 1
}
**return***f̂*^(^*^k^*^)^
**End algorithm**

As apparent, a CR *f̂*-being an average of input points *x_i_* ∈ *N^D^*-belongs to *N^C^* and is, reasonably, the best representative for all the points *x_i_* ∈ *s_x_* having *d_H_*(*x*, *f̂*) less or equal to the allowed defect number. It is worth noticing that *f̂* (and its quantized version *Q*(*f̂*)) may or may not be present in the input sequence *s_x_*.

With this formulation the algorithm quickly (i.e. within 3 ÷ 5 iterations) converges to a local minimum that can be arbitrarily far from the optimal solution. In fact, for sparse landscapes, the procedure does not redistribute points among classes in a global way, i.e. a shallow local minimum is likely to be found. However, an important observation is that, if a sufficient number of CRs is present, these will preferentially converge towards the high density regions of string space since the clusters act as attraction basins. If the number of classes *C* is too small, the method does not resolve the clusters at fine grain. Vice versa, If *C* is too large, the CRs interfere with each other and they converge towards regions which can be rather far from the cluster centroids.

The aim of our method is two-fold. On one hand, we wish to have an optimal number of CRs so that the clusters are resolved with controlled resolution. On the other hand, we wish to avoid getting trapped into some local minimum by driving the addition of new CRs in the neighborhood of the high density regions. Therefore, the algorithm is generalized to be adaptive in the number of classes *C*, and new CRs are inserted and optimized in the string space with some guiding principles.

### 2.2. Generation of new class representatives

We introduce the input parameter *m* as the number of mismatches allowed between the patterns and their CR. The procedure begins by inserting a small number of random, uniformly distributed, points in *N^C^* constituting a starting set of CRs.

The following iterative procedure describes how new CRs are inserted, and further optimized. The procedure is initiated by setting the counter *K =* 1.

1. For each class *α*, the number of class elements with Hamming distance larger than *m* defines the spread of the class, *S_α_*, given by
  (8) $$S_{\alpha }=\sum_{i\in \alpha }\Theta [d_{H}(x_{i},{\hat{x}}_{\alpha })-m]$$
  where the characteristic function is Θ(*x*) = 1 If *x*\*geq* and Θ(*x*) = 0 If *x* < 0.
2. A fraction (≈30%) of CRs of classes having largest spread {*S_α_*} are split into new CRs according to the following prescription. Let us define the column dispersion of the profile matrix as
  (9) $$D_{l}(x)=\sqrt{\frac{1}{4}\sum_{\alpha =A,C,G,T}{\left(\omega_{l,\alpha }(x)-\frac{1}{4}\right)}^{2}}$$
  where the term 1/4 within brackets is the mean of the column values.
  If all elements of a given column are close to 1/4 the column dispersion is minimum. Starting from each CR *f̂_α_* to be split, the new class representative *f̂_α_* *^n^* is generated with profile matrix *ω* (***f̂αn***) built as a copy of *ω*(***f̂αn***) except for the *K* columns presenting the 1st, …, *K*th smallest dispersions {*D_l_*} among all the columns *l =* 1, *n*. These columns are changed by setting equal to one the element having the second best rank among all letters of the column (the three remaining elements of the column are set to zero). The underlying idea is that, in the clusters with large spread, there are many points far from the CR and, by changing as above the columns with the least dispersion, we focus on the least fixed nucleotides. For this reason, we are quite sure that there are many points containing, in the select columns, the nucleotide individuated by the position of the second element with largest dispersion. In such a way, we attempt to create a new class with a partitioning very different from the previous CR, i.e. we try to generate a new non-overlapping class.
3. The new set of CRs is optimized through the LBG algorithm described in the previous section. All CRs that, after the new partitioning, are found to have empty classes are removed from the CR pool.
4. If the number of mutations *K* is equal to the length of the motif, *n*, and the number of CRs has not changed, then Exit;//we are not able to individuate new, not-empty classes.
  Else Ifthe number of CRs has not changed, Then *K* → *K* + 1;//try a stronger perturbation to the original CR.
  Compute the number *S_α_* of elements which have distances from the CR larger than *m*, and let *N_α_* be the total number of elements (*N_α_* = *L* − *N +* 1).
  If the ratio *S_α_*/*N_α_* is smaller than a given tolerance (≈10%) then Exit;//nearly all the points have been classified.
  Else *K* → *K* + 1 and Goto 1.

### 2.3. Refinement of classes

Once the generation of new class representatives terminates and the set of CRs has converged around the clustered regions of string space, the class elements are evaluated. The patterns encoded by the discretized profile matrices are taken as putative consensus patterns and a standard statistical analysis is performed.

However, we have noticed that the quality of results is significantly improved by further refining the class elements. This is done by considering, besides the Hamming distance (2), the edit distance as a further measure of similarity between strings. The edit distance relies on the alignment between two strings of (potentially) different length; its definition is based on dynamic programming [Jones and Pevzner, 2004] and reflects the minimum number of editing operations (mutations, insertions and deletions) needed to transform one string into another. In our implementation, each operation adds up a score of +1 to the edit distance. The metrics in *N^D^* is thus based on the generalized distance

(10) $$\tilde{d}(x,y)=\text{min}(d_{H}(x,y),d_{E}(x,y))$$

The new measure takes into account the case of two sequences, like *s*_1_ = *AGAGAG* and *s*_2_ = *GAGAGA*, having maximal *d_H_*, but being very similar (it is sufficient to delete one character from one string to produce a perfect match).

The class elements are re-defined by considering all elements with *d̃* ≤ *m* from the closest CRs. With this definition, we have two important notions to keep in mind. Firstly, the input parameter *m* which was used to guide the insertion of new CRs based on the Hamming metrics now takes care also of insertions and deletions. Secondly, intersections among classes are now admitted, i.e. each element can belong to one or more classes, depending if the distance from the respective CRs is smaller than *m*.

Furthermore, we have found that the optimized CRs are sensitive to the choice of the initial random CRs, and performances can be improved by resorting to multiple runs with different initial conditions. However, the search appears to be rather conclusive by cycling over a limited (of the order of 10) number of initial conditions.

### 2.4. Post-processing

In the statistical analysis of the over-represented patterns, we now specialize the search to the case of *K* biological input sequences. We consider the case in which one of the sequences may or may not contain any occurrence of the pattern.

The analysis of the class elements is performed by employing usual statistical indicators taken from the literature [Stormo, 2000; Pavesi et al. 2004]. By definition, a signal *P* is such if there exists a pattern of length *n* which is represented multiple times within the *m* allowed defects from the instances {*x_i_*}. The statistical importance of the signal *P* is described by two key quantities, the strength and the significance of the class representing *P* through its CR. The strength indicates the number of times the signal occurs in the text. The significance measures the degree of novelty of the set with respect to a background statistics.

The use of distinct indicators allows us to analyze in detail the statistical features of the class representatives. However, in practical applications, it is more desirable to combine these indicators into a single quantity. We will explore such possibility in a further extension of the work.

The three indicators we consider are

- the consensus, as a measure of the strength of the signal [Pavesi et al. 2004],
  (11) $$C_{P}=n*N_{P}-2\sum_{i=1}^{K}\tilde{d}(P,x_{i})I(P,x_{i})$$
- where *N_P_* is the number of sequences that contains, at least once, the signal *P.* Moreover, *d̃* (*P*, *x_i_*) is the generalized distance between the pattern and its best instance in the *i-*th sequence. Finally, *I*(*P*, *x_i_*) assigns the score +1 to every match and a penalty −1 to every mismatch between *P* and its best occurrence within the *i*-th sequence. Clearly, *C_P_* does not contain information about the statistical significance of the signal but only on the number of signals populating the class in the different DNA sets.
- the degree of dispersion of the signal is given by the relative entropy *S_P_*, defined in [Pavesi et al. 2004] and slightly modified to take into account the occurence probability of a given *n*-mer *x_i_*(*n*) = (*x_i_*, …, *x_i_* _+_ *_n_*_−1_).
  (12) $$S_{p}(x_{n})=\sum_{i=1}^{n-m_{o}+1}P_{r}[x_{i}(m_{o})]\text{log}\left(\frac{P_{r}[x_{i}(m_{o})]}{P_{b}[x_{i}(m_{o})]}\right)$$
  where *m_o_* is the order of the Markov model built with the available background sequences and *P_r_*[*x_i_*(*m_o_*)] is the frequency of occurrence of the sequence *x_i_*, …, *x_i_*_−m_o_ + 1_ obtained by averaging over the instances of the pattern. Moreover, *P_b_*[*x_i_*(*m_o_*)] is the frequency of occurrence of the *m_o_*-gram obtained over the instances of the pattern within a background sequence. The latter is estimated by using a Laplace sample-size correction to avoid underflows [Gelman et al. 2003].
- the deviation of the instances of the signal from its expected value provides a third important indicator
  (13) $$Z_{P}=\frac{N_{p}-E(P)}{\sigma (P)}$$
  where *N_p_* is the number of occurrences of *P*, *E*(*P*) is the expectation of *P*, and *σ*(*P*) is the standard deviation in the number of input *P* sequences.

The previous indicators are computed for each element of each class and are associated to each class representative as the ensemble average computed over all the class elements. The indicators are associated to the CRs and are used to perform a strong, not linear filtering on the classes produced through the processes described in Sections 2.1, 2.2 and 2.3. Let *I*_1_ and *I*_2_ be two of the three indicators (for instance, *I*_1_ = *C_P_* and *I*_2_ = *S_P_*). First of all, the classes are sorted on the basis of the *I*_1_ values associated to the CRs. In such a way, the elements in the head position are the strongest signals, while the tail classes represent the weakest signals. Following such an ordering, a fraction *α* of the weakest classes is discarded, generating a set of (1−*α*)*C* classes (the ones representing the strongest signals). After this filtering, we order again the (1−*α*)*C* classes on the basis of *I*_2_. Now, the elements in the head position are characterized by high statistical significance. Thanks to this approach, IMAGE identifies, as the best ranked solutions, the classes representing both significant and strong signals.

## 3. Discussion

The efficiency of IMAGE has been assessed by using the test-case provided by Tompa et al. [Tompa et al. 2005]. Such a benchmark has been recently used to promote a “contest” to survey the quality of different tools capable of predicting TF binding sites of bacterial and eukaryotic genomes.

The test runs as follows [Tompa, 2005]: the user is provided with an input data set containing a number of regulation regions related to different co-expressed genes, for a number of organisms, such as: human (file names with prefix *hm*), mouse (file names with prefix *mus*), *D. melanogaster* (file names with prefix *dm*), and *S. cerevisiae* (file names with prefix *yst*). The benchmark is, indeed, the composition of three different tests, each of them related to the same binding sites, but differing in the way the sequences outside the binding sites are constructed. In particular, the benchmark named “real” (file names with suffix *r*) has the binding sites in their real genomic promoter sequences. The benchmark named “generic” (file names with suffix *g*) has the binding sites merged in randomly chosen genomic promoter sequences from the same organism. The benchmark named “markov” (file names with suffix *m*) has the binding sites merged in sequences randomly generated according to a Markov chain of order 3 that was constructed from the promoter sequences of the same organism. It also provides the known regions which the tools should be able to identify.

We used all the provided fasta files as benchmarks for IMAGE. For each organism, a specific background file has been used consisting of intergenic sequences taken from [1]. IMAGE is a rather flexible software which allows to tune different input parameters in order to improve the quality of the search results. For example, IMAGE allows to select the motif lengths and the number of allowed mismatches. **Moreover, the possibility of reading the input sequence in its complementary form associated to the DNA second strand is straightforward and does not affect the results. However, in the following we will concentrate on one-way reading of the input sequence.** Although the software input parameters have not been optimized exhaustively for the contest under consideration, we have chosen a set of input parameters which produced results with greater statistical significance. The quality of results could be ameliorated in the future. Following [Pavesi et al. 2004], for all sequences different runs were performed by searching motifs of type (6, 1), (8, 2), (10, 3), (12, 4), where the symbol (*k*, *m*) means motifs of length *k* and allowing for at most *m* mismatches within the class. Among sites belonging to a class and overlapping along the sequence, only the patterns with minimum generalized distance (10) are further considered. All the predictions are sorted on the basis of the consensus *C_P_* (11) of which 80% of the top ranked CR are next ordered according to the relative entropy *S_P_* (12). The CR scoring the highest relative entropy represents the final motif resulting from the search.

**Figure 1:**
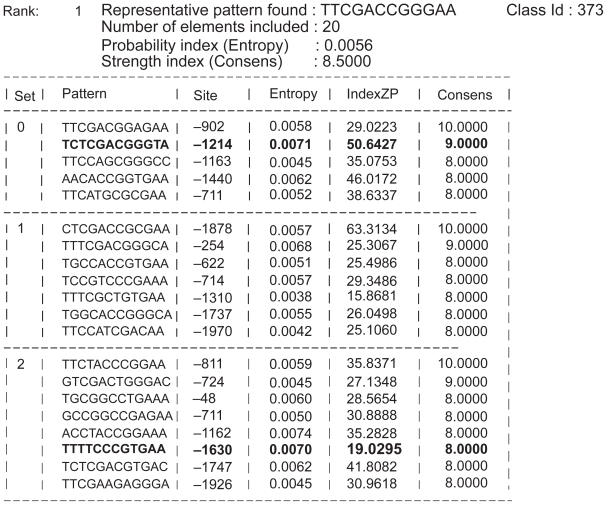
Part of the IMAGE tool output for *D. melanogaster* sequence *dm03g,* illustrating the detailed description of elements belonging to the best class representative found on the basis of the chosen probability indicator (*S_P_* in this case).

**Figure 2:**
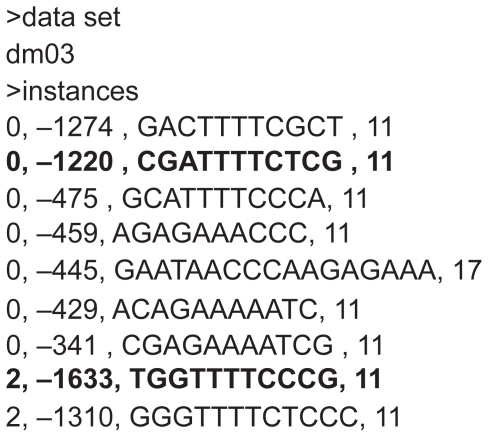
Known TF binding sites along *D. melanogaster* sequence *dm03g,* used in the assessment. The answers are retrieved from the Web site (http://bio.washington.edu/assessment/answer.txt).

Figure 1 illustrates part of the IMAGE output applied to the search of the *D. melanogaster* sequences of the benchmark (file *dm03g*), obtained by searching (10, 3) motifs. In the Table displayed in Figure 1, for each motif belonging to the reported class, the output provides the corresponding set, the found motif, the position along the sequence, the probability parameters (*S_P_* and *Z_P_*) and the number of matches with respect to the class representative (*C_P_*). For the same test case *dm03g*, Figure 2 displays the known motifs (i.e. the known “solution” to the problem), where the match between the predicted motifs (of Fig. 1) and the known motifs are reported in bold.

In order to qualify the performances of IMAGE, we have submitted our results to the analysis tool provided in the assessment web site [Tompa, 2005]. To this aim, the union of all predicted sites resulting from the highest ranked classes produced by the (6, 1), (8, 2), (10, 3) and (12, 4) searches were submitted for statistical evaluation.

The complete set of results of IMAGE is provided as Additional Material. The typical IMAGE output consists of:

- a summary of all input parameters,
- a table containing the first 20 top ranked class representatives, ordered on the basis of the selected probability indicators,
- a detailed description of the 20 top ranked classes with tables similar to that illustrated in Figure 1.

Moreover, the software analyzes and reports the correlation distance between different CRs. By specifying a cutoff distance between the position of pairs of putative sites, IMAGE evaluates the number of sequences, belonging to two different CRs, which are within that cutoff. Such a number identifies the correlation distance between the two CRs. CRs pairs are then sorted according to their correlation distance In this way, following the analysis present in the Co-bind software [Guhathakurta and Stormo, 2001], cooperative binding factors can be also visualized.

The validation of our method occurs through the estimate of different statistical indicators pertaining to the ability of selecting the correct DNA regions. These indicators can be constructed on the basis of the following quantities: the number of true positives (TP), true negatives (TN), false positives (FP) and false negatives (FN). The values of these quantities result from the comparison of the software output (Fig. 1) with the provided TF binding sites (Fig. 2). The quality of predictions are evaluated on the basis of the following indicators [Tompa et al. 2005]:

(14) $$xSn=\frac{TP}{\left(TP+FN\right)}$$

(15) $$xPPV=\frac{TP}{\left(TP+FP\right)}$$

(16) $$xSP=\frac{TN}{\left(TN+FP\right)}$$

(17) $$xPC=\frac{TP}{\left(TP+FN+FP\right)}$$

(18) $$\begin{array}{l} xCC=(TP\times TN-FN\times FP)\times \\ \frac{1}{\sqrt{\left(TP+FN)\times (TN+FP)\times (TP+FP)\times (TN+FN\right)}} \end{array}$$

where *Sn* is the Sensitivity, *PPV* the Positive Predicted Value, *SP* the Specificity, *PC* the Performance Coefficient and *CC* the Correlation Coefficient, as defined in [Tompa et al. 2005]. The prefix *x* stands for nucleotide (*n*) or site (*s*) if the indicator is evaluated at the nucleotide or at the site level; in the first case, the statistical values refer to the number of nucleotide positions present in the known and predicted sites. In the second case, in turn, the different values refer to the overlap between known and predicted sites (see [Tompa et al. 2005] for a thorough explaination of the statistical diagnostics). *nSn* (or *sSn)* corresponds to the sensitivity for nucleotide (or for site) and *nPPV* (or *sPPV*) represents the Positive Predictive Value. The first quantity expresses the fraction of known nucleotides (or sites) that are predicted, while the second evaluates the fraction of predicted nucleotides (or sites) that are known. *nSp* is the Specificity, defined at nucleotide level, *nPC* is the performance coefficient according to [Pevzner and Sze, 2000], and *nCC* corresponds to a correlation coefficient according to [Burset and Guig, 1996]. All these parameters and their calculations are further described by Tompa and coworkers [Tompa et al. 2005]. Finally, *sASP* is the average value between *sSn* and *sPPV*, and represents an average site performance.

**Figure 3:**
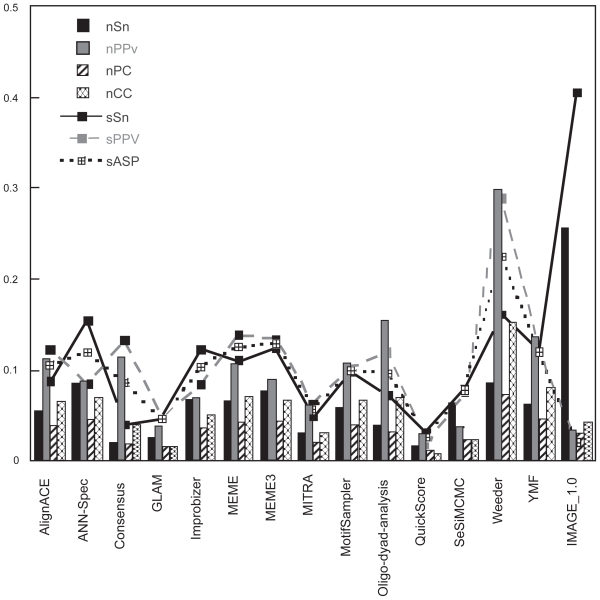
Summary of all statistical indicators for IMAGE, compared to those related to the other software results. Data relative to all other tools are reported in Figure 1 of [Tompa et al. 2005] and have been downloaded from [Tompa, 2005].

Figure 3 summarizes the values of all statistical indicators obtained for IMAGE as compared to the other software results, published in the cited assessment [Tompa et al. 2005]. As a general comment on the performances of IMAGE, the software extracts a large number of true positives (TP) with respect to the majority of the considered tools (presenting larger values of *sSn* and *nSn*). The large quantity of predicted regions implies, however, the presence of a large number of FPs which significantly reduces the overall score of indicators such as *xPPV.* As a results, IMAGE is capable of predicting a higher number of known nucleotides (larger values for nSn and sSn), but a lower number of predicted nucleotides (or sites) that are known (lower values of sPPV and nPPV). Moreover, the correlation (nCC) and performance (nPC) coefficients are sensibly lower than the corresponding values found for Weeder, but comparable to the ones obtained for MEME [Bailey and Elkan, 2000] and other tools.

In order to assess the capabilities of IMAGE, we have taken into consideration a different dataset, by spanning some of the crucial software control parameters used in the previous dataset. We have investigated a number of test cases which have been recently used to validate a proposed tool for the discovery of TF binding sites (tool GLAM, see http://zlab.bu.edu/glam/sup) [Frith et al. 2004]. Test sequences have been produced by inserting specific binding sites of a number of TF: 27 mammalian E2F (E2F), 35 bicoid (bcd), 27 Kriippel (Kr) and 25 mammalian estrogen response elements (ERE) binding sites, inserted within short sequences of nearly 50 bases. The performances of IMAGE are reported in Table 1 and compared with the contest dataset. Our method performs quite well by producing good values of the statistical indicators previously defined: in particular the sensitivity is rather high together with a substantial growth of the Positive Predicted Value, as compared to the data of Figure 3. As a result, the performance coefficient ranges between 0.27–0.329, depending on the sorting algorithm, which lends further confidence on the overall quality of IMAGE.

Overall, IMAGE presents the characteristics of an increased number of true positives, with respect to other tools present in the specialized literature. This, in the end, should be seen as an advantage: by combining the IMAGE output with further post-processing (such as, e.g. reference to an external TF database), our software should be able to improve the identification of putative binding sites and, thus, significantly reduce the number of false positives.

## Figures and Tables

**Table 1:** Statistical indicators for the contest and for the Glam test (E2F, bcd, Kr and ERE benchmarks—see text for details) obtained by sorting results via *C_p_* and via *S_p_* (as labels indicate).

Sorting	nSn	nPPV	nSp	nPC	nCC
Contest-*S_p_*	0.25635	0.033987	0.85596	0.030936	0.043827
GlamTest-*C_p_*	0.47511	0.39593	0.79949	0.27545	0.25792
GlamTest-*S_p_*	0.537890	0.459003	0.819235	0.329191	0.338292
